# Valence band offset of β-Ga_2_O_3_/wurtzite GaN heterostructure measured by X-ray photoelectron spectroscopy

**DOI:** 10.1186/1556-276X-7-562

**Published:** 2012-10-10

**Authors:** Wei Wei, Zhixin Qin, Shunfei Fan, Zhiwei Li, Kai Shi, Qinsheng Zhu, Guoyi Zhang

**Affiliations:** 1 State Key Laboratory of Artificial Microstructure and Microscopic Physics, School of Physics, Peking University, Beijing, 100871, People's Republic of China; 2 Key Laboratory of Semiconductor Materials Science, Institute of Semiconductors, Chinese Academy of Sciences, P.O. Box 912, Beijing, 100083, People's Republic of China

**Keywords:** β-Ga_2_O_3_/wurtzite GaN heterostructure, Band offset, X-ray photoelectron spectroscopy

## Abstract

A sample of the β-Ga_2_O_3_/wurtzite GaN heterostructure has been grown by dry thermal oxidation of GaN on a sapphire substrate. X-ray diffraction measurements show that the β-Ga_2_O_3_ layer was formed epitaxially on GaN. The valence band offset of the β-Ga_2_O_3_/wurtzite GaN heterostructure is measured by X-ray photoelectron spectroscopy. It is demonstrated that the valence band of the β-Ga_2_O_3_/GaN structure is 1.40 ± 0.08 eV.

## Background

GaN has been used in many applications including field-effect transistors and high-electron mobility transistors [1,2]. However, the power-handling capability of these devices is limited by the leakage current through the Schottky gate. To solve this problem, GaN-based metal-oxide-semiconductor (MOS) or metal-insulator-semiconductor structures are being widely investigated. Ga_2_O_3_ can be used as the gate dielectric medium for GaN-based MOS devices to suppress the gate leakage current [3]. Because gallium oxide can crystallize in monoclinic crystalline form (β-Ga_2_O_3_) in the process of fabricating a β-Ga_2_O_3_/GaN-based MOS structure, the β-Ga_2_O_3_ layer can be formed on GaN epitaxially. In this case, the β-Ga_2_O_3_ layer can be formed as an oxide (insulator) layer with a certain crystalline structure within the MOS structure. Apart from the crystalline quality of the Ga_2_O_3_ layer, the band parameters, such as band offsets, also play an important role in the current transport mechanism. These parameters determine the barrier for hole or electron transport across the interface. There is a similar influence to that on the current transport mechanism in a β-Ga_2_O_3_/GaN dual-color photodetector [4].

The growth and structural characterization of Ga_2_O_3_/GaN heterostructures by dry thermal oxidation on GaN have been reported extensively [5-7]. However, to date, the band alignment of the Ga_2_O_3_/GaN heterostructure has not yet been determined experimentally. In this paper, the Ga_2_O_3_/GaN heterostructures were fabricated by a thermal process method. Because the Ga_2_O_3_ can be grown epitaxially on GaN, the Ga_2_O_3_/GaN heterostructure with the epitaxial relationship between the Ga_2_O_3_ and GaN layers allows us to evaluate the band offset of the heterostructure. X-ray photoelectron spectroscopy (XPS) is a powerful tool for measurement of the valence band offsets (VBOs) of heterostructures. Experimental measurements of the VBO for the Ga_2_O_3_/GaN heterostructure by XPS were conducted, and the conduction band offset (CBO) was also calculated. These measurements are important for understanding the current transport mechanism of Ga_2_O_3_/GaN-based electronic devices.

## Methods

To measure the VBO values, three samples were used: a 6-μm-thick GaN layer grown on a c-plane sapphire substrate as sample I, a 200-nm-thick Ga_2_O_3_ layer on a GaN template as sample II, and an approximately 5-nm-thick Ga_2_O_3_ layer on a GaN template as sample III. The GaN samples were grown on c-plane (0001) sapphire substrates by metal organic chemical vapor deposition (MOCVD). Trimethylgallium and blue ammonia were used as the Ga and N sources, respectively, for MOCVD growth. In our experiments, the GaN sample was thermally oxidized in a 600 ml/min oxygen ambient for 10 min at 900°C, and an approximately 5-nm-thick Ga_2_O_3_ layer was obtained on the GaN surface. The GaN sample was thermally oxidized in the same condition but for 8 h, and a 200-nm-thick Ga_2_O_3_ layer was obtained on the GaN surface. The GaN thin-film surface has a root-mean-square (RMS) roughness of 0.3 nm as revealed by AFM. The RMS roughness of the approximately 5-nm-thick Ga_2_O_3_ layer surface is 2.7 nm. The Ga_2_O_3_ thickness was measured by XPS, and the Ga_2_O_3_ crystal structures were characterized using an X-ray diffraction (XRD) apparatus. The XRD measurements were carried out using an X'Pert Pro MPD diffractometer (CuKα radiation; PANalytical B.V., Almelo, The Netherlands) with an X'Celerator detector. The XRD patterns were then refined using the HighScore Plus (PANalytical B.V.) and FullProf software packages. The XPS measurements were performed at room temperature using a PHI Quantera SXM instrument (Physical Electronics GmbH, Ismaning, Germany) with AlKα (hv = 1486.6 eV) as the X-ray radiation source, which had been carefully calibrated based on the work function and the Fermi level (*E*_F_). The total energy resolution of this XPS system is approximately 0.5 eV, and the accuracy of the observed binding energy is within 0.03 eV after careful calibration [8]. Before taking the measurements, the XPS apparatus is calibrated by fitting to the Fermi edge of an Ar^+^-bombarded silver sample. The accuracy of the observed binding energy (368.26 ± 0.03 eV for Ag 3*d*_5/2_) is within 0.03 eV. When the sample is measured, a large number of electrons are excited and emitted from the sample, so the sample is always positively charged and the resulting electric field can affect the measured kinetic energy of the photoelectrons. A low-energy electron flood gun was used to achieve charge compensation, and all of the XPS spectra were calibrated using the C1*s* peak at 284.8 eV from contamination to compensate for the charge effect. In order to avoid the pernicious effect of surface contamination on the XPS measurement of the Ga_2_O_3_/GaN heterojunction, an Ar^+^ bombardment with a voltage of 1 kV at a low sputtering rate of 0.5 nm/min was carried out.

## Results and discussion

**Figure 1:**
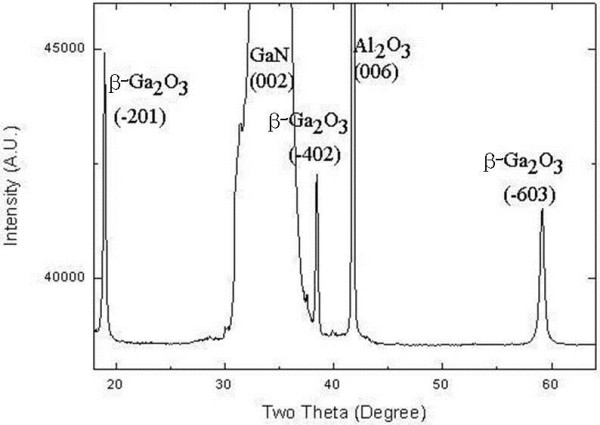
XRD pattern of β-Ga_2_O_3_ formed on a GaN template for sample III.

According to the results of the XRD measurements, peaks from the (−201), (−402), and (−603) planes of β-Ga_2_O_3_ and the (002) plane of wurtzite GaN were observed in sample III, as shown in Figure 1. The epitaxial relationships were found to be (−201) β-Ga_2_O_3_//(002) wurtzite GaN.

From the theory first introduced by Kraut [9], for the β-Ga_2_O_3_/wurtzite GaN heterostructure, the VBO (Δ*E*_v_) value can be calculated from the following formula:

(1) $$\begin{array}{l} \Delta E_{V}=\Delta E_{\mathrm{CL}}-\left(E_{O1\text{s}}^{{\text{Ga}}_{2}{\text{O}}_{3}}-E_{\mathrm{VBM}}^{{\text{Ga}}_{2}{\text{O}}_{3}}\right)\\ \;+\left(E_{N1s}^{\text{GaN}}-E_{\mathrm{VBM}}^{\text{GaN}}\right) \end{array}$$

where $\Delta E_{\mathrm{CL}}=E_{O1\text{s}}^{{\text{Ga}}_{2}{\text{O}}_{3}}-E_{N1s}^{\text{GaN}}$ is the energy difference between the N1*s* and O1*s* core levels in GaN and Ga_2_O_3_, which can be measured from the sample Ga_2_O_3_/GaN heterostructure that was prepared by growing the approximately 5-nm β-Ga_2_O_3_ layer on the GaN template. $E_{O1\text{s}}^{{\text{Ga}}_{2}{\text{O}}_{3}}-E_{\mathrm{VBM}}^{{\text{Ga}}_{2}{\text{O}}_{3}}$ is the energy difference between Ga_2_O_3_ O1*s* and the valence band maximum (VBM) in the Ga_2_O_3_ thick film, and *E*_*N*1*s*_^GaN^ − *E*_*VBM*_^GaN^ is the energy difference between GaN N1*s* and the VBM in the GaN thick film. Similarly, the Ga 3*d* spectra of both Ga_2_O_3_ and GaN can also be used to calculate the VBO of the Ga_2_O_3_/GaN heterostructure. The related data are summarized in Table 1.

**Table 1:** XPS core-level spectra curve-fitting results and VBM positions used to calculate VBO of the Ga_2_O_3_/GaN heterostructure

**Sample**	**State**	**Binding energy** (**eV**)	**Bonding**	**FWHM** (**eV**)
Ga_2_O_3_	Ga 3*d*	20.22	Ga-O	1.35
	O1*s*	531.15	Ga-O	1.58
	VBM	3.10		
GaN	Ga3*d*	19.89	Ga-N	1.35
	N1*s*	397.18	Ga-N	1.18
		395.61	Ga Auger	1.89
		393.44	Ga Auger	2.95
	VBM	2.22		
Ga_2_O_3_/GaN	Ga3*d*	20.56	Ga-O	1.23
		19.57	Ga-N	1.03
	O1*s*	531.27	Ga-O	1.72
	N1*s*	396.93	Ga-N	1.75
		395.36	Ga-Auger	2.36
		393.19	Ga-Auger	3.02

FWHM, full width at half maximum.

**Figure 2:**
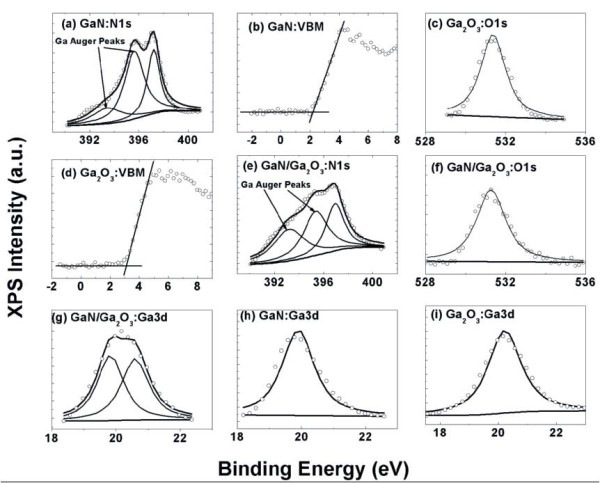
**Core levels and VBE spectra.** (**a**) Core level of N1*s* recorded in a 6-μm-thick GaN film. (**b**) The VBE spectra from the 6-μm-thick GaN film. (**c**) Core level of O1*s* recorded in a 200-nm-thick Ga_2_O_3_ film. (**d**) The VBE spectra of the 200-nm-thick Ga_2_O_3_ film. (**e**) Core level of N1*s* recorded on the Ga_2_O_3_/GaN heterostructure sample. (**f**) Core level of O1*s* recorded on the Ga_2_O_3_/GaN heterostructure sample. (**g**) Core level of Ga3*d* recorded on the Ga_2_O_3_/GaN heterostructure sample. (**h**) Core level of Ga3*d* recorded in a 6-μm-thick GaN film. (**i**) Core level of O1*s* recorded in a 200-nm-thick Ga_2_O_3_ film.

Figure 2a,b,h gives the core level of N1s, the valence band edge (VBE) spectra, and the core level of Ga3*d* recorded from a 6-μm-thick GaN film, respectively. Figure 2c,d,i displays the core level of O1*s*, the VBE spectra, and the core level of Ga3*d* recorded from a 200-nm-thick Ga_2_O_3_ film, respectively. Figure 2e,f,g shows the core level of N1*s*, O1*s*, and Ga3*d* recorded from the Ga_2_O_3_/GaN heterostructure sample, respectively. All core level peaks were fitted using a Shirley background and Voigt (mixed Lorentzian-Gaussian) line shapes. The VBM positions in the VB spectra were determined by linear extrapolation of the leading edges of the VB spectra to the base lines to account for any instrument resolution-induced tails. The peak parameters and the VBM positions from Figure 2 are shown in Table 1 for clarity [10].

In Figure 2a,e, the N1*s* peaks in both the GaN and Ga_2_O_3_/GaN samples have quite asymmetrical shapes and consist of three components. The two lower binding energy components are associated with the Ga Auger peaks [11,12], and the higher binding energy component is considered to be from Ga-N bonding.

As shown in Table 1, the energy difference between N1*s* and the VBM of the GaN film (*E*_*N*1*s*_^GaN^ − *E*_*VBM*_^GaN^) is 394.96 eV, the energy difference between O1*s* and the VBM of the Ga_2_O_3_ film $\left(E_{O1\text{s}}^{{\text{Ga}}_{2}{\text{O}}_{3}}-E_{\mathrm{VBM}}^{{\text{Ga}}_{2}{\text{O}}_{3}}\right)$ is 528.05 eV, and the energy difference between the N1*s* and O1*s* core levels in GaN and Ga_2_O_3_, $\Delta E_{\mathrm{CL}}=E_{O1\text{s}}^{{\text{Ga}}_{2}{\text{O}}_{3}}-E_{N1s}^{\text{GaN}}$, is 134.34 eV. The Ga_2_O_3_/GaN VBO is therefore 1.25 ± 0.08 eV for the O1*s*-N1*s* combination.

**Figure 3:**
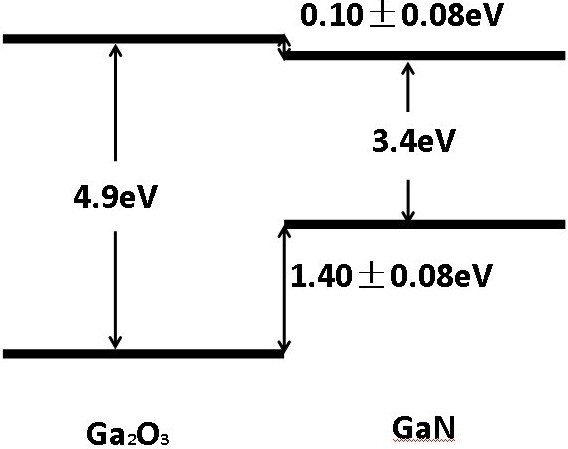
Energy band diagram of Ga_2_O_3_/GaN heterostructure at room temperature.

As shown in Table 1, the energy difference between N1*s* and the VBM of the GaN film (*E*_*N*1*s*_^GaN^ − *E*_*VBM*_^GaN^) is 394.96 eV, which is consistent with the data reported by Sato et al. [13]. Similarly, the energy difference between Ga3*d* and the VBM of the GaN film (*E*_*Ga*3*d*_^GaN^ − *E*_*VBM*_^GaN^) is 17.67 eV, which agrees with the results of Craft et al. [14]. Similarly, the energy difference between Ga3*d* and the VBM of the Ga_2_O_3_ film $\left(E_{Ga3d}^{{\text{Ga}}_{2}O_{3}}-E_{\mathrm{VBM}}^{{\text{Ga}}_{2}O_{3}}\right)$ is 17.12 eV, which is in accordance with the results reported by Hui et al. [15]. Table 2 lists the VBO values determined by substituting the values in Table 1 into a similar formula to Equation 1 using different combinations of the XPS core levels. The average Ga_2_O_3_/GaN VBO is 1.40 ± 0.08 eV for the four combinations. The CBO can then be calculated using the formula $\Delta E_{c}=E_{g}^{{\text{Ga}}_{2}{\text{O}}_{3}}-E_{g}^{\text{GaN}}-\Delta E_{v}$. The bandgap of Ga_2_O_3_ is 4.90 eV, as reported elsewhere [16]. Similarly, the bandgap of GaN is 3.40 eV [17]. The energy band diagram of the Ga_2_O_3_/GaN heterostructure is therefore determined at room temperature, with a CBO of 0.10 ± 0.08 eV, as shown in Figure 3.

**Table 2:** VBOs calculated for the Ga_2_O_3_/GaN heterostructure using different combinations of the XPS core levels

	**Ga3*d***	**N1*s***
Ga3*d*	1.54	1.47
O1*s*	1.25	1.32

The errors in the VBOs are ±0.08 eV.

## Conclusions

In summary, β-Ga_2_O_3_ films have been grown on a wurtzite GaN underlayer with an epitaxial relationship of β-Ga_2_O_3_ (−201)//wurtzite GaN (002). The VBO of the β-Ga_2_O_3_ (−201)/wurtzite GaN (002) heterostructure has been measured by XPS to be 1.40 ± 0.08 eV, with a corresponding CBO of 0.10 ± 0.08 eV from the calculation. Accurate determination of the VBO of GaN/Ga_2_O_3_ is critical for the design and application of Ga_2_O_3_/GaN-based electronic and optoelectronic devices [18].

## Competing interests

The authors declare that they have no competing interests.

## Authors' contributions

WW did the experiment, studied the data, and got the result. ZQ, SF, and GZ revised the paper including spell errors and grammar. ZL, KS, and QZ instructed how to analyze the data. All authors read and approved the final manuscript.
